# Recognition of Elicitors in Grapevine: From MAMP and DAMP Perception to Induced Resistance

**DOI:** 10.3389/fpls.2019.01117

**Published:** 2019-09-18

**Authors:** Marie-Claire Héloir, Marielle Adrian, Daphnée Brulé, Justine Claverie, Sylvain Cordelier, Xavier Daire, Stéphan Dorey, Adrien Gauthier, Christelle Lemaître-Guillier, Jonathan Negrel, Lucie Trdá, Sophie Trouvelot, Elodie Vandelle, Benoit Poinssot

**Affiliations:** ^1^Agroécologie, Agrosup Dijon, CNRS, INRA, Univ. Bourgogne, Univ. Bourgogne Franche-Comté, Dijon, France; ^2^Unité RIBP EA 4707, SFR Condorcet FR CNRS 3417, University of Reims Champagne-Ardenne, Reims, France; ^3^UniLaSalle, AGHYLE Research Unit UP 2018.C101, Rouen, France; ^4^Laboratory of Pathological Plant Physiology, Institute of Experimental Botany, the Czech Academy of Sciences, Prague, Czechia; ^5^Laboratory of Plant Pathology, Department of Biotechnology, University of Verona, Verona, Italy

**Keywords:** *Vitis vinifera*, innate immunity, defense responses, Pattern Recognition Receptor (PRR), Microbe-Associated Molecular Pattern (MAMP), Damage-Associated Molecular Pattern (DAMP), Induced Resistance (IR)

## Abstract

In a context of a sustainable viticulture, the implementation of innovative eco-friendly strategies, such as elicitor-triggered immunity, requires a deep knowledge of the molecular mechanisms underlying grapevine defense activation, from pathogen perception to resistance induction. During plant-pathogen interaction, the first step of plant defense activation is ensured by the recognition of microbe-associated molecular patterns, which are elicitors directly derived from pathogenic or beneficial microbes. *Vitis vinifera*, like other plants, can perceive elicitors of different nature, including proteins, amphiphilic glycolipid, and lipopeptide molecules as well as polysaccharides, thanks to their cognate pattern recognition receptors, the discovery of which recently began in this plant species. Furthermore, damage-associated molecular patterns are another class of elicitors perceived by *V. vinifera* as an invader’s hallmark. They are mainly polysaccharides derived from the plant cell wall and are generally released through the activity of cell wall–degrading enzymes secreted by microbes. Elicitor perception and subsequent activation of grapevine immunity end in some cases in efficient grapevine resistance against pathogens. Using complementary approaches, several molecular markers have been identified as hallmarks of this induced resistance stage. This review thus focuses on the recognition of elicitors by *Vitis vinifera* describing the molecular mechanisms triggered from the elicitor perception to the activation of immune responses. Finally, we discuss the fact that the link between elicitation and induced resistance is not so obvious and that the formulation of resistance inducers remains a key step before their application in vineyards.

## Introduction

*Vitis vinifera* cultivars, cultivated worldwide for the production of table grape and wines, are susceptible to various pathogens, such as insects, viruses, bacteria, phytoplasmas, fungi, and oomycetes. The last two groups of microorganisms can rapidly and strongly impact the yield and the quality of the harvest. For example, the oomycete *Plasmopara viticola* and the ascomycete *Erysiphe necator*, the causal agents of downy and powdery mildews, respectively, infect leaves but also inflorescences and young green berries, while *Botrytis cinerea*, a necrotrophic fungus causing gray mold, affects berries during ripening. These devastating diseases are currently managed mainly through repeated treatments with synthetic fungicides for grape protection. However, the intensive use of chemicals has a negative impact on environment and human health and contributes to the selection of resistant pathogenic strains (Casida, 2009; Nanni et al., 2016). Thus, in the context of the integrated pest management, the implementation of a sustainable viticulture requires alternative/complementary strategies to chemical treatments. Among them, a recognized approach for crop protection is the stimulation of natural plant defense to trigger induced resistance (IR) (Walters et al., 2013; Delaunois et al., 2014).

**Figure 1:**
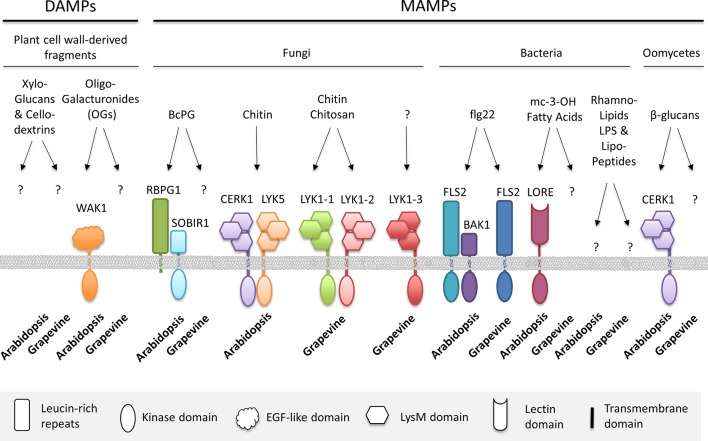
Microbe- and damage-associated molecular patterns (MAMPs, DAMPs) recognized by grapevine or *Arabidopsis* pattern recognition receptors (PRRs), which trigger plant immunity. BAK1, brassinosteroid-insensitive 1 (BRI1)–associated receptor kinase 1; BcPG, *B. cinerea* polygalacturonase; CERK1, chitin elicitor receptor kinase 1; EGF, epidermal growth factor; FLS2, flagellin sensing 2; LORE, Lipooligosaccharide-specific reduced elicitation; LPS, lipopolysaccharide; LYK, lysin motif-containing receptor-like kinase; OGs, oligogalacturonides; RBPG1, responsiveness to botrytis polygalacturonases 1; SOBIR1, suppressor of BIR1; WAK1, wall-associated kinase1.

Plants detect pathogen attack through the perception of conserved microbial signatures, called microbe-associated molecular patterns (MAMPs) or host-derived damage-associated molecular patterns (DAMPs), thanks to plant plasma membrane pattern recognition receptors (PRRs) (Boutrot and Zipfel, 2017). PRRs are generally receptor-like kinases (RLKs) or receptor-like proteins (RLPs) with an extracellular domain for MAMP/DAMP recognition (Trdá et al., 2015). MAMPs can be microbial structural components such as bacterial flagellin, lipopolysaccharides (LPSs), peptidoglycans (PGNs), rhamnolipids (RLs), fungal chitin, or oomycete β-glucans but also secreted toxins or enzymes like fungal xylanase or endopolygalacturonase (**Figure 1**). DAMPs are endogenous danger signals, such as oligogalacturonides (OGs) or cutin monomers, released from degraded plant cell wall or cuticle, respectively (Boller and Felix, 2009). The MAMP/DAMP perception triggers a complex cascade of signaling events, including ion fluxes such as Ca^2+^ influx, the production of reactive oxygen species (ROS) and nitric oxide, and the activation of mitogen-activated protein kinases (MAPKs). These early events induce a massive transcriptional reprogramming of primary and secondary metabolisms. The induction of specific defense-related genes leads to the synthesis of (i) pathogenesis-related (PR) proteins, including hydrolytic enzymes (e.g., β-1,3-glucanases or chitinases), which degrade pathogen cell wall; (ii) antimicrobial compounds like phytoalexins; (iii) compounds involved in cell wall reinforcement; and, in some cases, (iv) proteins involved in the hypersensitive response (HR), a form of programmed host cell death (Garcia-Brugger et al., 2006). Altogether, these defense reactions represent the MAMP-triggered immunity (MTI), which is finely regulated by phytohormones (salicylic acid [SA], jasmonic acid [JA], and ethylene [ET]) to finally prevent or delay pathogen infection, thus ensuring a basal plant resistance to pathogens.

The possibility of activating plant immunity using MAMPs or DAMPs led to the development of IR strategy to protect crops against pathogens. Indeed, the application of elicitors mimics a pathogen attack, stimulating basal plant defenses and thus leading to plant resistance, at least in controlled conditions (Wiesel et al., 2014). Elicitors can be purified MAMPs or DAMPs, molecules displaying similar structures or even extracts derivate from microorganisms or plants, and can have a biological or synthetic origin (Walters and Fountaine, 2009). For grapevine, several elicitors of various origins or structural patterns have been studied so far, and their mode of action has been well characterized (Delaunois et al., 2014). Some elicitors are able to induce a good level of resistance against pathogens in controlled conditions. For example, the β-glucan laminarin and its sulfated derivative (PS3) were shown to protect grapevine against *P. viticola* (Aziz et al., 2003; Trouvelot et al., 2008). Chitin and its deacetylated derivative chitosan act as MAMPs (Brulé et al., 2019) and induce grapevine resistance against *P. viticola*, *E. necator*, and *B. cinerea* (Trotel-Aziz et al., 2006; Aziz et al., 2007). Recently, it has been shown that a mixture of chitosan and OGs (COS-OGA) protects grapevine against powdery mildew in vineyards (van Aubel et al., 2014). Nevertheless, it is known that the efficacy of IR in the field is variable, depending on various biotic and abiotic factors (Adrian et al., 2012; Delaunois et al., 2014). Therefore, it is crucial to get a better understanding of IR mechanisms and to identify associated molecular markers.

In this review, we focus on the current knowledge about MAMPs/DAMPs which trigger immune responses in grapevine and also on their cognate PRRs in this plant. Moreover, we discuss the relation between the elicitation of immune responses and the actual IR, as well as the perspectives to improve the effectiveness of resistance inducers in a perspective of field application.

## Active MAMPs and DAMPs Perceived By *Vitis Vinifera*

### The ***B. cinerea*** Endopolygalacturonase 1 (BcPG1) and its Activity-Derived Products (OGs) Are Distinct Interconnected Elicitors That Activate Independently Grapevine Immune Responses

Bacterial or fungal necrotrophic phytopathogens secrete numerous plant cell wall–degrading enzymes (CWDEs) to break down the cell wall polymers, such as cellulose, hemi-cellulose, and pectin, and invade host tissues. The degradation of host cell wall then contributes to the development of soft rots or molds. Among necrotrophs, *B. cinerea*, an opportunistic plant pathogenic fungus able to cause rot in many plant tissues, including in grapevine, produces a variety of pectinases, including exo- and endopolygalacturonases, pectin methylesterases, pectin, and pectate lyases (ten Have et al., 2002; Zhang and van Kan, 2013). In particular, its genome encodes for six endopolygalacturonases (BcPG1-6), the expression of which varies depending on plant tissues (ten Have et al., 1998; ten Have et al., 2001). The biochemical characterization of their enzymatic activity and the production of deletion mutants of *BcPG1* and *BcPG2* demonstrated their capacity to produce tissue collapse and necrosis as well as their role in *B. cinerea* virulence on tomato, apple, broad bean, and *Arabidopsis thaliana* (hereafter *Arabidopsis*) (ten Have et al., 1998; Kars et al., 2005). The availability of a set of endoPGs with slightly different characteristics in terms of substrate specificity might enable the pathogen to hydrolyze a larger spectrum of pectin types, originating from different host species.

The degradation of pectin by polygalacturonases leads to the release of intermediate products, called OGs, which have been shown to act in vivo as DAMPs, protecting plants from infection by necrotrophic pathogens (Benedetti et al., 2015). OGs are α-1,4-linked galacturonosyl residues with different degrees of polymerization (DP) and esterification, which may influence their elicitor activity. Indeed, based on several transcriptomic studies, long OGs (DP > 10) have long been thought to be the only efficient pectin fragments for triggering plant defense responses (Moscatiello et al., 2006; Ferrari et al., 2007; Denoux et al., 2008). However, although short OGs suppress defense responses in wheat (Moerschbacher et al., 1999), they can also induce a strong defense gene expression in other plants, such as *Arabidopsis*, potato, and tomato (Simpson et al., 1998; Norman et al., 1999). Recently, a comparison of gene expression reprogramming, induced by both long (DP > 10) and short (DP3) OGs, revealed actually a great similarity of the response, in qualitative and quantitative terms, confirming that both long and short OGs are potentially biologically active, at least in *Arabidopsis* (Davidsson et al., 2017).

The formation of OGs during plant-pathogen interactions is promoted by polygalacturonase-inhibiting proteins (PGIPs) (Kalunke et al., 2015). These plant cell wall proteins selectively and partially inhibit PGs produced by pathogenic microorganisms allowing the accumulation of OGs, as intermediate reaction products, which are active elicitors of plant defense responses. In particular, expression of the grapevine VvPGIP1 in tobacco was shown to improve plant resistance against *B. cinerea*, through the partial inhibition of some fungal PGs, including BcPG1 (Joubert et al., 2006). On the other hand, OGs can be inactivated by berberine bridge enzyme (BBE)–like proteins, which act as specific OG oxidases (Benedetti et al., 2018). Through such *in vivo* OG oxidase (OGOX) activity, oxidized OGs become less active as defense inducers and less susceptible to hydrolysis by fungal PGs. It has been proposed that this mechanism may prevent the deleterious excessive OG accumulation, which may compromise plant growth and resistance through cell death induction, when reaching high concentrations (Cervone et al., 1987). Although OGOX activity has still to be identified in grapevine, it is reasonable to assume that OG turnover may be controlled in the same way to ensure grapevine growth-immunity trade-off.

**Figure 2:**
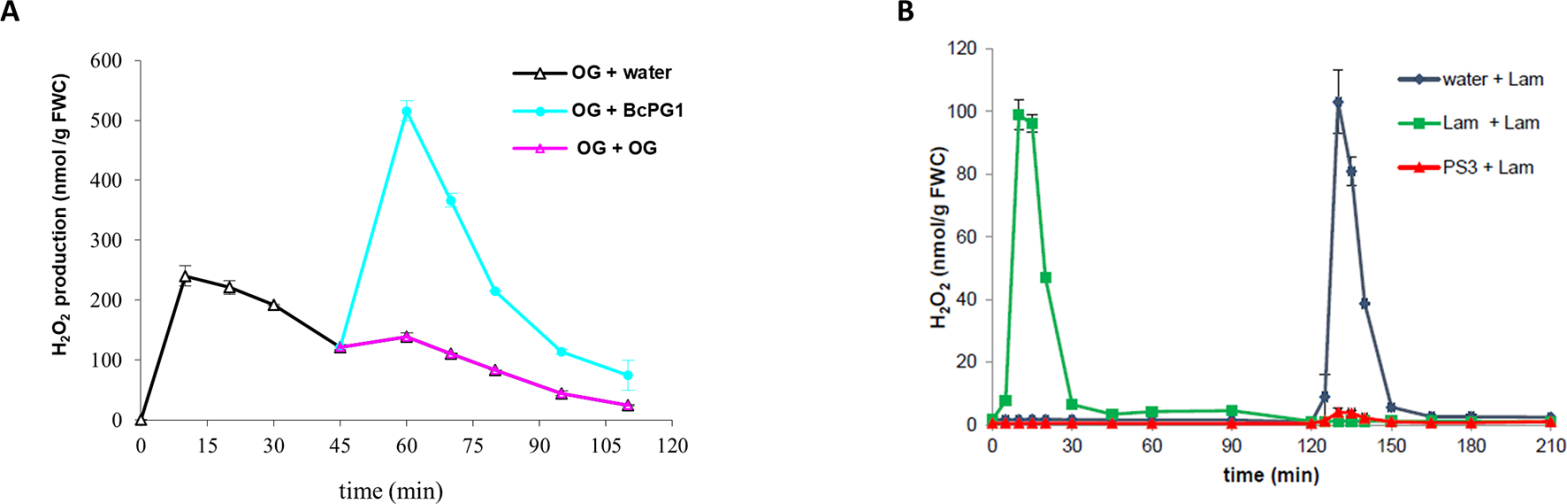
Desensitization experiments can differentiate MAMPs/DAMPs perceived by independent or common receptors in grapevine. **(A)** Desensitization experiments reveals that BcPG1 and OGs are perceived differently by grapevine cells as the H_2_O_2_ production is abolished after two successive treatments with OG (pink) but not when BcPG1 (cyan) succeeds to a first treatment with OGs (black). Cells were first treated at time 0 with OGs (200 µg/ml; black), washed three times between 30 and 45 min with fresh medium, then treated a second time with OGs (200 µg/ml; pink), BcPG1 (0.2 µg/ml; cyan), or water (black). H_2_O_2_ production was determined using chemiluminescence of luminol as described by Poinssot et al. (2003). **(B)** Refractory state experiments revealed that the two β-glucan derivatives (PS3 and Lam) might be perceived by the same receptor in grapevine cells as a first treatment with PS3 abolishes the oxidative burst normally elicited by laminarin. Cells were first treated at time 0 with water (black), laminarin (Lam, 4 mg/ml; green), or PS3 (4 mg/ml; red); washed three times between 90 and 120 min with fresh medium; and then treated a second time with laminarin (4 mg/ml). H_2_O_2_ production was determined using chemiluminescence of luminol as described by Gauthier et al. (2014). Data are means ± SE of duplicates, representative of three independent experiments. FWC, fresh weight of cells.

Some strains of *B. cinerea* are unable to develop on grapevine, likely due to the induction of strong plant defense responses and the incapacity of the fungus to counteract such defense mechanisms (Derckel et al., 1999). Accordingly, an eliciting activity was found in the filtrate of the T4 strain of *B. cinerea*, nonpathogenic on grapevine, and, surprisingly, attributed to the protein BcPG1 (Poinssot et al., 2003). The protein BcPG1 was also more abundant in the nonpathogenic A336 mutant of *B. cinerea*, impaired in plant colonization capacity, and compared to the wild-type pathogenic strain Bd90, and this larger BcPG1 amount was correlated with the induction of a strong oxidative burst in grapevine cells (Kunz et al., 2006). It was thus assumed that the overproduction of BcPG1 may boost plant defense reactions against the mutant strain, thus contributing to its reduced pathogenicity. Interestingly, BcPG1 elicitor activity was not related to its enzymatic activity, i.e., from release of OGs from grapevine cell wall, but rather relied on the recognition of the protein itself (Poinssot et al., 2003). In particular, besides the overall lower intensity of defense responses induced by OGs compared to BcPG1, grapevine cells pre-treated with OGs were not desensitized to the successive application of BcPG1, and vice versa, indicating the existence of two different receptors/pathways leading to defense induction (**Figure 2A**). Moreover, BcPG1 elicitor and enzymatic activities were discriminated by inhibiting selectively one or the other through different protein treatments (Poinssot et al., 2003). This study thus demonstrated that BcPG1 functions as a MAMP and that particular protein patterns may be responsible for defense induction, as previously reported for other CWDEs, such as the 22-kDa fungal protein ethylene-inducing xylanase (EIX), which *per se* functions as an elicitor, independently of its enzymatic activity (Enkerli et al., 1999; Furman-Matarasso et al., 1999; Rotblat et al., 2002). The challenging aspect in the elucidation of plant defense mechanism induction is the identification of perception systems, which have been often characterized using the model plant *Arabidopsis*, allowing genetic studies. Wall-associated kinases (WAKs) have been proposed for a long time as candidates for the perception of OGs (**Figure 1**). These proteins possess a typical intracellular Ser/Thr kinase domain and an apoplastic domain containing several epidermal growth factor (EGF)–like repeats interacting with the cell wall. The role of WAK RLKs in plant immunity has been demonstrated in several plant species, including *Arabidopsis*, rice, maize, and more recently tomato and wheat (Diener and Ausubel, 2005; Zhang et al., 2005; Li et al., 2009; Rosli et al., 2013; Hurni et al., 2015; Zuo et al., 2015; Delteil et al., 2016; Harkenrider et al., 2016; Zhang et al., 2017; Saintenac et al., 2018; Yang et al., 2019). In *Arabidopsis*, WAKs are encoded by five tightly linked and highly similar genes and several reports demonstrated in particular the capacity of WAK1 to bind OGs *in vitro* through the N-terminal ectodomain (Wagner and Kohorn, 2001; Decreux et al., 2006; Cabrera et al., 2008). Moreover, *WAK*1 was the only member of the gene family to be up-regulated in response to OGs treatment (Denoux et al., 2008). To overcome the limits of reverse genetics due to redundancy and lethality of WAK silencing (Wagner and Kohorn, 2001), an elegant domain swap approach, using the construction of a chimeric receptor, demonstrated the function of WAK1 as a receptor of OGs and its role in defense response induction in *Arabidopsis* (Brutus et al., 2010). WAK1-mediated OG-induced responses were shown to be then negatively regulated by two WAK1 interactors, namely, the glycin-rich protein GRP-3 and a kinase-associated protein phosphatase (KAPP), which likely contribute to phase out the plant immune response (Gramegna et al., 2016).

On the other hand, by exploiting the capacity of *B. cinerea* PGs to induce different levels of necrosis formation in different *Arabidopsis* accessions, a map-based cloning strategy combined with comparative and functional genomics allowed the identification of the RLP RBPG1 (RESPONSIVENESS TO BOTRYTIS POLYGALACTURONASES 1) as endoPG receptor in *Arabidopsis* (Zhang et al., 2014). Lacking a cytoplasmic functional domain, the RBPG1 function as PRR relies on its interaction with the RLK SOBIR1 (for SUPPRESSOR OF BIR1). The formation of the RBPG1/SOBIR1 heterodimer, unaffected by the BcPG3 ligand, likely ensures intracellular signal transduction through SOBIR1 kinase activity (**Figure 1**). RBPG1 not only interacts with BcPG3 but also recognizes multiple active and inactive PGs from *B. cinerea*. Since the response of *Arabidopsis* to PGs appears different from that reported in other plant species (Kars et al., 2005; Joubert et al., 2007; Zhang et al., 2014), the identification of homologs of AtRBPG1, or other RLPs, should be carried out in *V. vinifera* to characterize its perception system for BcPG1.

Following OGs and BcPG1 perception, several studies reported a detailed description of the induced signaling events using pharmacological, biochemical, and genetic approaches. Both elicitors trigger an increase of cytosolic Ca^2+^ concentration ([Ca^2+^]_cyt_) (Lecourieux et al., 2002; Vandelle et al., 2006). Ca^2+^ increase controls in turn the oxidative burst, which involves the activity of NADPH oxidases (Vandelle et al., 2006; Galletti et al., 2008) but also the production of nitric oxide (NO) (Vandelle et al., 2006; Rasul et al., 2012) by a NO synthase activity that still needs to be further characterized (Jeandroz et al., 2016). Independently of the above-mentioned physiological events, both BcPG1 and OGs activate two MAPKs related to AtMPK3 and AtMPK6 (Vandelle et al., 2006; Denoux et al., 2008; Galletti et al., 2011). Finally, as later events, both elicitors induce a set of defense-related genes and the production of phytoalexins, contributing to plant resistance.

The secretion of CWDEs by *B. cinerea* occurs constitutively. In particular, *Bcpg1* (together with *Bcpg2*) is among the earliest expressed fungal genes during the infection of tomato leaves, suggesting that the corresponding enzyme may play a role in early stages of the infection process (ten Have et al., 2001). Since the activation of PRR-mediated immunity aims at inhibiting pathogen development at early stages of infection (Melotto et al., 2006; Zeng and He, 2010), the early BcPG1 expression fits well with a possible rapid perception of the pathogen by grapevine cells. Moreover, its crucial role in pathogenicity makes it an indispensable element for the pathogenic fungus, and this supports further plant adaptation to recognize this protein that may remain stable in fungus genome to maintain its virulence, concept at the basis of the gene-for-gene/guard and decoy models (van der Hoorn and Kamoun, 2008). We could thus assume a scenario in which BcPG1 may represent the main elicitor of defense reactions. However, in case of perception failure by host cells, for instance in absence of the proper receptor, the enzymatic activity of BcPG1, regulated by PGIPs, would lead to the production of active OGs able to trigger grapevine immunity. Although less intense, this alternative defense layer may ensure the induction of a general mechanism of resistance to fight against *B. cinerea* infection and would be secured by the further inactivation of OGs by OGOXs once reached a concentration threshold.

### The Cell Wall–Derived Xyloglucans and Cellodextrins Are New DAMPs That Elicit Immune Responses in Grapevine

It has been previously reported that xyloglucan oligosaccharides, the main component of hemicellulose, play a role in cell wall structure (Takeda et al., 2002; Whitney et al., 2006), in the development and the regulation of plant growth in different plant species (Fry et al., 1993; Vargas-Rechia et al., 1998) and can also act as a storage polysaccharide to provide energy for the emerging seedlings (dos Santos et al., 2004). Interestingly, xyloglucans were recently discovered as new DAMPs in grapevine and *Arabidopsis* (Claverie et al., 2018).

In grapevine, xyloglucans elicit a broad range of defense responses, including the activation of two MAPKs and the production of the phytoalexin resveratrol, correlated with the induction of the expression of defense genes, involved in particular in the phenylpropanoid pathway and resveratrol biosynthesis. Importantly, xyloglucans are able to protect grapevine detached leaves against *B. cinerea* (Claverie et al., 2018). Besides grapevine, xyloglucans also trigger the phosphorylation of MAPKs, defense gene expression, and callose deposition, through the callose synthase PMR4 in *Arabidopsis* (Luna et al., 2011), and confer resistance against *B. cinerea* and the biotrophic oomycete *Hyaloperonospora arabidopsidis*. The use of *Arabidopsis* mutants allowed to provide evidence that xyloglucan-mediated resistance against *B. cinerea* involves different defense pathways, including camalexin, SA, JA, and ethylene (Claverie et al., 2018). Compared to OGs, the immune responses induced by xyloglucans are strikingly different in terms of kinetics and strength of the response or activated immune responses. Indeed, xyloglucans do not elicit H2O2 production neither in grapevine nor in *Arabidopsis* cells, while OGs trigger resistance against *B. cinerea* independently of SA, JA, and ethylene (Ferrari et al., 2007). These results suggest that different DAMPs could operate through different signaling pathways.

Xyloglucan oligomers are characterized by a β-1,4 glucan backbone resembling that of cellodextrins (CDs). CDs and cellobiose are cellulose-derived oligomers from the cell wall that also act as DAMPs, eliciting [Ca^2^+]_cyt_ variations, defense gene activation, and protection against pathogens in *Vitis vinifera* and *Arabidopsis* (Aziz et al., 2007; Souza et al., 2017). As shown for OGs, the elicitor activity of CDs can be impaired by a member of the BBE-like protein family in *Arabidopsis*, namely, the cellodextrin oxidase (CELLOX), via the oxidation of their reducing end, likely as a general mechanism of BBE-like proteins to avoid oligosaccharide hyperaccumulation (Locci et al., 2019). Moreover, a poly(A)ribonuclease controls the cellotriose-based interaction between *Arabidopsis* and the plant-growth-promoting endophyte *Piriformospora indica* suggesting a major role of this enzyme in regulating the outcome of this beneficial interaction (Johnson et al., 2018).

The structure of the oligosaccharide elicitors also plays an important role in their biological activity. For example, xyloglucans used in our study were mainly constituted of a β-1,4-glucan backbone associated with xylosyl-, galactosyl-, and fucosyl-type branching, with a DP of 7 (Claverie et al., 2018). The order and the type of substituents depend on the plant species, the cell type, and the developmental state of the cell (Pauly and Keegstra, 2016). As indicated above for OGs, the biological activity of oligosaccharides is known to be dependent on their DP and decoration pattern. Accordingly, only CDs with a DP > 7 are able to induce a strong production of H2O2, the overexpression of defense genes associated with an increase in chitinase and glucanase activities, finally leading to an IR against *B. cinerea *(Aziz et al., 2007). The future comparison of immune responses triggered by xyloglucans possessing different carbohydrate decorations would therefore deserve further investigations and might lead to interesting applications for crop protection.

Overall, the activation of immune responses in grapevine and *Arabidopsis* strengthens the notion that these two plant species can perceive hemicellulose and cellulose–derived oligomers and that at least one cognate receptor exists. As *Arabidopsis* perceive xyloglucans and cellobiose, it should be envisaged to first isolate their PRR(s) through a genetic approach, and then identify their orthologs in *V. vinifera*.

### The Grapevine LysM Receptors VvLYK1-1 and VvLYK1-2 Recognize Chitin and Chitosan Fragments to Elicit Similar Immune Responses

Chitooligosaccharides (COS), especially chitin and chitosan, are representative MAMPs known to induce immune responses in a wide range of plant species such as *Arabidopsis* (Miya et al., 2007; Cao et al., 2014) or rice (Hayafune et al., 2014). Chitin, a linear homopolymer of β-1,4 *N*-acetylglucosamine (GlcNAc), is a major structural component of fungal cell walls, Crustacean shells, and the exoskeleton of insects and nematodes. Chitosan, the deacetylated chitin derivative produced by chitin deacetylases, is also a naturally occurring polysaccharide, albeit less common. Chitosan can be notably found in some fungal species such as *Cryptococcus neoformans* (Baker et al., 2007). Since the 1980s, chitin and its deacetylated product chitosan have been used for crop farming as biopesticides, biofertilizers, for seed coating formulation, and agricultural film (El Hadrami et al., 2010; Hadwiger, 2013; Trouvelot et al., 2014). Both molecules are known to induce the biosynthesis of several antimicrobial compounds, namely, phytoalexins (Vasiukova et al., 2001), callose (Ren et al., 2001), and lignin (Barber et al., 1989). Many studies have reported that COS enhance plant defenses against bacteria (Rabea et al., 2003; Tikhonov et al., 2006), fungi (Park et al., 2002; Trotel-Aziz et al., 2006) and nematodes (Khalil and Badawy, 2012; Nunes da Silva et al., 2014).

Chitin hexa-, hepta-, or octamers are recognized by the lysin motif (LysM)-RLK chitin elicitor receptor kinase 1 (CERK1/LYK1) and LysM-containing receptor-like kinase5 (LYK5) in *Arabidopsis* (Miya et al., 2007; Wan et al., 2008; Petutschnig et al., 2010; Cao et al., 2014), by the complex OsCERK1/chitin elicitor-binding protein (OsCEBiP) in rice (Kaku et al., 2006; Shimizu et al., 2010) or by their ortholog complex MtLYK9/MtLYR4 in legumes such as *Medicago truncatula* (Bozsoki et al., 2017). Plant LysM domain proteins are required for chitin-binding and signaling and serve as modules mediating plant immunity and stopping pathogen infection. For instance, in line with AtCERK1 function in chitin recognition in *Arabidopsis*, the corresponding knockout mutant *Atcerk1/lyk1*, which completely lost its ability to respond to the elicitor, is more susceptible to the fungal pathogen *Alternaria brassicicola* and to the bacterial pathogen *Pseudomonas syringae* (Miya et al., 2007; Gimenez-Ibanez et al., 2009). In grapevine, a phylogenetic analysis highlighted three LysM proteins VvLYK1-1, VvLYK1-2, and VvLYK1-3, among 15, which are putative orthologues of the *Arabidopsis* AtCERK1/LYK1 and the rice OsCERK1 (Brulé et al., 2019). The functional complementation of the *Arabidopsis Atcerk1* mutant with the three grapevine orthologs of *AtCERK1/LYK1* demonstrated that the constitutive expression of *VvLYK1-1* or the inducible expression of *VvLYK1-2*, but not VvLYK1-3, restored COS-induced immune responses (Brulé et al., 2019). These results suggested that the same PRRs, VvLYK1-1, and VvLYK1-2 are involved in COS-triggered immunity in grapevine (**Figure 1**). Moreover, *VvLYK1-1* expression in the *Atcerk1* mutant background was demonstrated to restore non-host resistance against the grapevine powdery mildew *E. necator*.

Downstream *V. vinifera* receptor activation, chitosan elicits phytoalexins, chitinase, and glucanase activities leading to resistance against *B. cinerea* and *P. viticola* (Aziz et al., 2006; Trotel-Aziz et al., 2006). More precisely, hexamers of chitin and chitosan elicit the phosphorylation of MAPKs, phytoalexin production, and expression of defense genes, finally inducing resistance against the necrotrophic fungus *B. cinerea* and the obligate biotrophic oomycete *P. viticola* (Brulé et al., 2019). However, chitin hexamer do not induce any oxidative burst, contrary to what is observed in *Arabidopsis* (Miya et al., 2007).

### The Flagellin-Derived Peptides Flg22 From Pathogenic or Beneficial Bacteria Recognized by the Grapevine VvFLS2 Receptor Do Not Elicit Similar Immune Responses

Flagellin, the main building protein of eubacterial flagella, is a potent defense elicitor in different plant species and is one of the best studied MAMPs. Plants perceive flagellin mainly through the N-terminal conserved epitope of 22 amino acids, called flg22. Flg22 peptide from *Pseudomonas aeruginosa* is a commonly used MAMP to study the responses to flagellin in a broad variety of plant species (Boller and Felix, 2009). Using *V. vinifera* cell suspensions, flg22 was shown to be also an active MAMP in grapevine (Trdá et al., 2014).

The plant PRR responsible for flagellin/flg22 perception is the leucine-rich repeat receptor like kinase (LRR-RLK) flagellin sensing 2 (FLS2) (Gomez-Gomez and Boller, 2000; Chinchilla et al., 2006). FLS2 was first identified in *Arabidopsis* (AtFLS2) (Gomez-Gomez and Boller, 2000), and functional FLS2 orthologues were further shown to be conserved within the plant kingdom (Boller and Felix, 2009). Among them, a clear orthologue of *AtFLS2* was identified in the grapevine genome, namely, *VvFLS2*, encoding a protein displaying a conserved domain structure. The functionality of VvFLS2 as a grapevine flagellin receptor (**Figure 1**) was demonstrated through the successful complementation of the corresponding *Arabidopsis* mutant *fls2*, which successfully restored flg22-induced H2O2 production (Trdá et al., 2014), as previously shown for the rice OsFLS2 or the tomato LeFLS2 (Takai et al., 2008; Mueller et al., 2012). At molecular level, the perception of flg22 in grapevine by VvFLS2 triggers common rapid and transient signaling responses, such as an increase in free [Ca^2+^]_cyt_, the phosphorylation of two MAPKs with relative molecular masses of 45 and 49 kDa and an oxidative burst. Later, flg22 perception activates the expression of a set of defense genes (e.g., genes encoding the acidic chitinase Chit4c and the protease inhibitor PR6). Finally, flg22-elicited responses leads to a partial resistance of grapevine against *B. cinerea* (Trdá et al., 2014).

Previous studies have reported different recognition specificities between FLS2 from tomato and *Arabidopsis* (Felix et al., 1999; Bauer et al., 2001; Sun et al., 2006; Robatzek et al., 2007; Mueller et al., 2012). Interestingly, expressing *VvFLS2* in the *fls2* background of *Arabidopsis* conferred a flagellin responsiveness profile specific to grapevine. Indeed, while different flg22 peptides, derived from different bacteria, exhibited comparable biological activities in *Arabidopsis*, they showed distinct eliciting activities in grapevine, and such species-specific differences in flg22 perception were caused, at least in part, by the nature of *Arabidopsis* and grapevine FLS2 proteins. *Burkholderia phytofirmans* is a plant growth-promoting rhizobacterium (PGPR) naturally associated with grapevine (Ait Barka et al., 2000; Compant et al., 2005; Lo Piccolo et al., 2010) which modifies grapevine metabolism thus promoting an improved plant growth, a better tolerance to *B. cinerea* infection as well as to cold stress (Ait Barka et al., 2000; Ait Barka et al., 2006; Fernandez et al., 2012; Theocharis et al., 2012). Furthermore, it can also trigger grapevine defense responses, including extracellular medium alkalization and defense gene expression, suggesting that it is potentially perceived *via* MAMP detection (Bordiec et al., 2011). Accordingly, the flagellin purified from *B. phytofirmans* induces *PR1* gene expression in *Arabidopsis*. Similarly, flg22 from this endophytic bacterium (*Bp* flg22) triggers immune responses also in grapevine, but weaker than the responses triggered by flg22 peptides derived from the pathogenic bacteria *Xanthomonas campestris* (*Xc* flg22) (**Table 1**) or *P. aeruginosa* (*Pa* flg22) (Trdá et al., 2014). In addition, *Bp* flg22-triggered gene expression is very low and transient compared with the long-lasting effect of *Xc* flg22 and *Pa* flg22 treatment, and some genes—for instance, *PR6* or the grapevine SA marker gene *17.3* (Bordiec et al., 2011), are not significantly activated by *Bp* flg22 treatment. Finally, unlike *Xc* flg22, *Bp* flg22 does not significantly inhibit grapevine plantlet growth (Trdá et al., 2014). While *B. phytofirmans* is naturally colonizing grapevine, it can colonize *Arabidopsis* only under laboratory conditions (Poupin et al., 2013; Zuniga et al., 2013), and it was therefore proposed that alterations in the *Bp* flg22 sequence might represent a successful adaptation of *B. phytofirmans* to avoid recognition by the host VvFLS2 and activation of immune system.

**Table 1:** Intensity of the immune responses, in *Vitis vinifera* and *Arabidopsis thaliana*, following treatment with 1 µM of flagellin-derived flg22 epitopes derived from beneficial or pathogenic bacteria. ++, highly induced; +, induced; +/−, weakly induced; −, not induced; PGPR, plant growth-promoting bacteria. Results from Trdá et al., 2014.

Bacteria	Type	Flagellin epitope	*Vitis vinifera*			*Arabidopsis thaliana*		
			H_2_O_2_ production	Defense gene expression	Plant growth inhibition	H_2_O_2_ production	Defense gene expression	Plant growth inhibition
*B. phytofirmans*	PGPR	Bp flg22	+/−	+/−	+/−	++	++	++
*X. campestris*	Pathogen	Xc flg22	++	++	++	++	++	++
*A. tumefaciens*	Pathogen	At flg22	−	−	−	−	−	−

Key amino acids described as crucial for flg22-eliciting activity (Felix et al., 1999; Bauer et al., 2001; Sun et al., 2006) are unchanged in *Bp* flg22. Nevertheless, three amino acid substitutions between the most active peptide in grapevine *Xc* flg22 and *Bp* flg22 are sufficient to explain this different sensitivity. The pathogenic bacterium responsible for crown gall disease, *Agrobacterium tumefaciens*, also possesses 12 mutations in flg22 avoiding the FLS2 recognition and the subsequent immune responses (**Table 1**). On the other hand, the fact that AtFLS2 and VvFLS2 do not similarly perceive different flg22 epitopes, especially *Bp* flg22, suggests that VvFLS2 has evolved to distinguish flagellin originating from the grapevine-associated PGPR *B. phytofirmans*. Therefore, VvFLS2 and/or flagellin from *B. phytofirmans* may have undergone evolutionary changes allowing the adapted endophytic bacterium to colonize its natural host plants without inducing a strong MTI. Of interest, not only *B. phytofirmans* evades perception by grapevine receptor, but it also seems to overcome flg22-induced MTI to efficiently colonize grapevine plants. Indeed, the application of the strong elicitor *Xc* flg22 to the roots of grapevine plantlets during the first stage of bacterization did not affect the process of plant colonization by *B. phytofirmans*. VvFLS2 thus represents the first example of a characterized receptor that differentially recognizes flg22 epitopes from a PGPR and plant-pathogenic bacteria, suggesting an evolutionary mechanism of grapevine innate immunity evasion by its beneficial endophytic bacterium *B. phytofirmans*.

### Rhamnolipids, LPS, and Cyclic Lipopeptides Are Recognized by Grapevine and Trigger-Induced Resistance

Amphiphilic glycolipid and lipopeptide compounds have been recently identified as effective inducers of immune response in grapevine. Among them, bacterial RLs are the best characterized. Within the first hour after treatment, RLs from *P. aeruginosa* and *Burkholderia plantarii* trigger strong early signaling events in grapevine cell suspensions as well as defense markers, including genes encoding PR proteins or enzymes involved in oxylipin and phytoalexin biosynthesis pathways, and a programmed cell death reminiscent of animal apoptosis at high concentrations (Varnier et al., 2009). Furthermore, RLs potentiate defense responses induced by other elicitors and induce a local resistance of grapevine against the necrotrophic fungus *B. cinerea* (Varnier et al., 2009; Vatsa et al., 2010) but also an effective protection of *Brassica napus* and *Arabidopsis* toward fungal diseases (Sanchez et al., 2012; Monnier et al., 2018). In *Arabidopsis*, RL-mediated resistance involves different signaling pathways that depend on the type of pathogen. While JA is essential for the resistance to *B. cinerea*, the ET-mediated signaling pathway is involved in *Arabidopsis* resistance to the oomycete *H. arabidopsidis* and the bacterium *Pseudomonas syringae* pv. *tomato* (Sanchez et al., 2012). SA-dependent plant defenses are primed by RLs following pathogen infections suggesting that SA also participates to the restriction of these pathogens. How RLs are perceived by plant cells is still unclear. A recent report suggested that RLs could insert within the lipid bilayer of plant membranes leading to a moderate membrane destabilization influenced by the nature of the phytosterols. The resulting changes in lipid dynamics could have a direct impact on plant defense induction (Monnier et al., 2019). In grapevine, RLs trigger a very strong Ca^2+^ influx (Varnier et al., 2009), with concentrations of free cytosolic calcium reaching values higher than the concentrations detected after the treatment of tobacco cell suspensions with the potent elicitor cryptogein (Lamotte et al., 2004). This Ca^2+^ signature seems to be very characteristic when compared with the signatures described for other elicitors (Garcia-Brugger et al., 2006) and could be related to the potential destabilization of grapevine cell membranes. Synthetic RL structures that induce a plant immune response are also able to interact with lipids from plant membrane models, suggesting that the perception of these molecules could involve a lipid-driven process (Nasir et al., 2017; Luzuriaga-Loaiza et al., 2018).

LPSs are complex amphiphilic glycolipids composed of a lipidic part (lipid-A), a conserved oligosaccharidic core, and an O-polysaccharidic part with variable length and composition (OPS) (Caroff and Karibian, 2003). They are present in the outer bilayer of the plasma membrane of most Gram-negative bacteria. While the lipid-A and the oligosaccharidic core are relatively well conserved, the OPS is chemically and structurally very heterogeneous among species. OPS is composed of polymers of several monosaccharide repeat units, and its variability depends on bacterial species (Lerouge and Vanderleyden, 2002). While the lipid-A is embedded in the outer membrane, the OPS is oriented outward of the membrane, thus being directly at the interface between the bacterium and the host during the interaction. The OPS plays an essential role in stress resistance, protecting bacteria against hostile environment and is considered as an important virulence factor for pathogenic bacteria during the colonization of their host (Clifford et al., 2013; Di Lorenzo et al., 2016). Phytopathogenic bacteria lacking OPS are generally considered less viable inside the host plant and are therefore expected to be non-virulent. For example, OPS mutants of *Erwinia amylovora*, *Ralstonia solanacearum*, *Xanthomonas axonopodis* pv. *citri*, or *Xylella fastidiosa* are significantly less virulent than their respective wild-type strains (Berry et al., 2009; Petrocelli et al., 2012; Clifford et al., 2013; Li et al., 2014). In contrast, host plants have developed perception systems of these LPSs leading to the induction of defense mechanisms to counter the invasion of pathogenic bacteria (Ranf et al., 2016). LPSs are considered as MAMPs and induce defensive responses, including ROS production, in several plant species such as *Arabidopsis*, tobacco, rice, or grapevine (Braun et al., 2005; Silipo et al., 2005; Desaki et al., 2006; Rapicavoli et al., 2018). In grapevine, *Agrobacterium vitis* and *Xylella fastidiosa*, two Gram-negative pathogenic bacteria, cause crown gall and Pierce’s disease, respectively. *A. vitis* LPS extracts infiltrated into grapevine internodes protect the plant from tumor appearance following *A. vitis* infection (Alexandrova et al., 2000). Similarly, LPSs purified from *X. fastidiosa* induce a rapid production of ROS in grapevine leaf disks. Interestingly, OPS-deficient *X. fastidiosa* bacteria cause a higher production of ROS in vitro and in vivo compared to wild-type bacteria (Rapicavoli et al., 2018). Grapevine plants pre-treated with LPSs from the OPS-deficient *X. fastidiosa* strain showed a strongly attenuated development of the symptoms caused by *X. fastidiosa* in comparison with grapevine plants pre-treated with purified LPSs from wild-type *X. fastidiosa* strain (Rapicavoli et al., 2018). The presence of the OPS therefore delays the immune perception of *X. fastidiosa* by grapevine cells, probably by masking the MAMP motifs on the cell surface. The OPS is also likely involved in bacterial virulence, especially in biofilm formation and motility, and therefore related to its ability to enter and spread within the host (Kutschera and Ranf, 2019). In *Arabidopsis*, it was recently shown that the lectin receptor S-domain receptor kinase AtLORE (LIPOOLIGOSACCHARIDE-SPECIFIC REDUCED ELICITATION, **Figure 1**) recognizes the medium chain 3-hydroxy fatty acid brick present in the lipid A part of LPSs but does not sense lipid A or LPSs themselves (Kutschera et al., 2019). Phylogenetically, this receptor seems to be specific of the Brassicaceae family (Ranf et al., 2015). Lipid-A from *Pseudomonas* seems also perceived by *Arabidopsis* as it induces a late oxidative burst, presumably not driven by the LORE receptor (Shang-Guan et al., 2018). This LORE-independent perception suggests that LPSs may be perceived by distinct perception mechanisms leading to the induction of the plant immune system. In rice, the perception of LPSs rather involves the OsCERK1 receptor, which also senses the lipid-A (Desaki et al., 2018). Bioinformatics and genetic approaches looking for homologous LORE-like or CERK1-like receptors might allow the identification of receptors involved in LPS perception by grapevine cells.

Lipopeptides produced by bacteria are also amphiphilic compounds perceived by plants as MAMPs (Ongena and Jacques, 2008). *Bacillus subtilis* produces three main families of cyclic lipopeptides (LPs), namely, surfactins, iturins, and fengycins. Purified surfactin, mycosubtilin (iturin family), and plipastatin (fengycin family) are sensed by grapevine plant cells (Farace et al., 2015). Although surfactin and mycosubtilin stimulate grapevine innate immune responses, they differentially activate early signaling pathways and defense marker genes. Plipastatin perception by grapevine cells only results in early signaling activation. Gene expression analysis suggested that mycosubtilin activates SA and JA signaling pathways, whereas surfactin mainly induces SA signaling. Interestingly, only surfactin and mycosubtilin treatments result in a local IR against *B. cinerea* in grapevine leaves. Challenge with specific *B. subtilis* strains overproducing surfactin and mycosubtilin also led to a slightly enhanced stimulation of grapevine defense responses compared with a wild-type strain of the bacteria (Farace et al., 2015). *B. subtilis* strain GLB191 confers protection to grapevine against downy mildew in controlled conditions and in the vineyard (Zhang et al., 2017). Recently, GLB191 supernatant was shown to be also highly active, with a double effect: oomycide and defense elicitor (Li et al., 2019). The supernatants of GLB191 mutants affected in the production of fengycin and/or surfactin loose partial or total activity, suggesting that both LPs contribute to the activity of GLB191 supernatant against downy mildew. As for RLs, the recognition mechanism of LPs is not fully understood. Mycosubtilin and fengycin are known to interact with biomimetic membrane lipids, in particular with phospholipids and sterols (Maget-Dana and Ptak, 1990; Deleu et al., 2005, Deleu et al., 2008). Studies on surfactin showed that the LP structure strongly impacts the immune response (Jourdan et al., 2009). The reduced activity of some surfactin homologues indicates that surfactin perception is dictated by structural clues in both the acyl moiety and cyclic peptide parts. Surfactin immune signatures suggest that these molecules could interact with membranes without irreversible pore formation but in a way sufficient to induce disturbance or transient channeling in the plasma membrane. This phenomenon could, in turn, activate a biochemical cascade of molecular events leading to defensive responses (Jourdan et al., 2009). Surfactins with long chains are more effective than the shortest ones. Repeated challenges with surfactin do not result in a refractory state (desensitization), suggesting that the sensing of the molecule does not involve a high-affinity protein receptor (Henry et al., 2011). Moreover, surfactin is able to target the lipid fraction of the plant plasma membrane, and long-chain surfactins are more active. Altogether, as for RLs, it is proposed that the recognition of these amphiphilic elicitors could rely on a lipid-driven process, leading to the perturbation of the membrane structure, and resulting in the activation of the immune response. Interestingly, this lipid-driven process seems to be less specific than the perception by PRRs as it could take place in several plant species, including grapevine. However, a dichotomy between monocots and dicots cannot be excluded as it has been shown for the perception of the toxin necrosis and ethylene-inducing peptide 1–like protein (NLP) by sphingolipid receptors (Lenarčič et al., 2017).

### Sulfation of the Laminarin β-Glucan Improves Its Resistance-Inducing Activity

The eliciting activity of the β-glucan oligosaccharides laminarin and its sulfated derivative PS3 have been studied in details. Laminarin is naturally produced by the brown algae *Laminaria digitata* and is a reserve carbohydrate of oomycetes (Bacic et al., 2009). It has an average DP of 25–33 glucose units and up to three single β-glucose branches at position 6 (Klarzynski et al., 2000). PS3 (phycarin sulfated 3) was obtained by chemical sulfation of laminarin and has a degree of sulfation of 2.4 (Ménard et al., 2004). In plants and mammals, the presence of sulfates is crucial for the biological activity of oligosaccharides, suggesting that chemical sulfation of oligosaccharides can improve their biological properties. Studies conducted with tobacco plants reported that, compared to laminarin, its sulfated derivative PS3 triggers a stronger immunity against tobacco mosaic virus (Ménard et al., 2004). Similarly, PS3 induces a stronger grapevine resistance against *P. viticola* compared to laminarin (Gauthier et al., 2014). In tobacco, Klarzynski et al. (2000) have shown that, while laminarin elicits phenylalanine ammonia lyase (PAL) activity, di-, tri-, and tetramers of β-1,3-glucan are inactive. In a structure-activity analysis, Ménard et al. (2004) demonstrated that the sulfate residues and a minimum β-1,3-glucan chain length (DP 5) are essential for PS3 activity in tobacco. Interestingly, PS3 and laminarin differ by their capacity to trigger (i) early grapevine defense responses and (ii) a stress-responsive transcriptome. Indeed, conversely to laminarin that elicits canonical early responses like cytosolic [Ca^2+^] variations, H2O2 production, MAPK activation, and plasma membrane depolarization, PS3 only induces a long-lasting plasma membrane depolarization. Furthermore, transcriptional changes observed in response to both β-1,3-glucans present similarities but differ in the intensity of transcriptome expression. Indeed, several genes are up-regulated by both β-1,3-glucans, but PS3 triggers a variation in the global transcriptional reprogramming 3.9-fold higher (Gauthier et al., 2014). Among the genes induced by PS3, many genes encode proteins involved in carbohydrate catabolism providing energetic nucleotides (e.g., ATP) and reduced cofactors (e.g., NADH and NADPH). These cofactors are particularly important for the activity of enzymes involved in defense responses. For instance, the NADPH is used by the respiratory burst oxidase homologue (RBOH) to produce an intense ROS production at the site of pathogen infection (Gauthier et al., 2014). This observation might also explain why sulfation of laminarin improved laminarin-IR against *P. viticola* in grapevine, with a HR-like profile (Trouvelot et al., 2008). Moreover, our results revealed that, compared to laminarin, PS3-IR against *P. viticola* in grapevine was correlated with the priming of defense genes, SA and H2O2 productions, callose deposition, and HR-like cell death. These various responses have been shown to be dependent on anionic channel activity (Gauthier et al., 2014). This activity has already been demonstrated to play key role in the establishment of the HR in tobacco (Wendehenne et al., 2002; Gauthier et al., 2007).

To assess whether PS3 and laminarin are perceived by a common receptor, H2O2 production assay was used to determine whether successive additions of PS3 and laminarin would result in a desensitized state. As expected, grapevine cells treated twice with laminarin were unable to respond to the second treatment (**Figure 2B**). Interestingly, the pre-treatment with PS3 prevented grapevine cells to respond to the subsequent addition of laminarin (**Figure 2B**). These data suggest that PS3 and laminarin are recognized by a common receptor and that sulfation of laminarin elicits the activation of different signaling cascades, leading to an enhanced immunity correlated to a higher resistance. Recently, the non-branched β-1,3-glucan hexamer has been shown to trigger *Arabidopsis* immune responses which were impaired in the *cerk1* mutant, suggesting that CERK1 might be a co-receptor for the β-1,3-glucan perception in this plant species (Mélida et al., 2018).

## Discussion

### Finding New VvPRRs and Investigating Their Spatiotemporal Expression in Grapevine

As described above, several elicitors of different nature, MAMPs or DAMPs, have been shown so far to efficiently induce immune responses in grapevine. Nevertheless, the discovery of grapevine PRRs began only recently and some information are still missing to fully understand the mechanisms of perception of these elicitors by grapevine cells. Based on the actual knowledge in *Arabidopsis*, it is highly probable that a grapevine ortholog for BRASSINOSTEROID-INSENSITIVE 1–ASSOCIATED RECEPTOR KINASE 1 (VvBAK1) co-receptor might also exist in grapevine to form the functional receptor complex with the flg22 receptor VvFLS2 (**Figure 1**). Similarly, a co-receptor of the AtCERK1/LYK1 orthologues VvLYK1-1 and VvLYK1-2 might also exist in grapevine. However, the bioinformatics predictions are more complicated because the grapevine genome contains two putative genes encoding orthologs of the rice OsCEBIP. Moreover, grapevine possesses 13 other genes encoding putative VvLYKs, the function of which is still unknown and including two orthologues of the *Arabidopsis* AtLYK5, known as the high-affinity binding domain of the chitin receptor complex (Cao et al., 2014). Interestingly, the expression of *VvCEBIP1* is induced by chitin treatment in grapevine cells (Trdá, 2014), while two *VvLYK5* genes are induced by the necrotrophic fungus *B. cinerea* during berry infection (Brulé et al., 2019). Concerning the *B. cinerea* PGs, the production of recombinant BcPG1 in the heterologous system *Pichia pastoris* was not very efficient (Kars et al., 2005), thus rendering the identification of its cognate receptor in grapevine very difficult. Nevertheless, since the *Arabidopsis* receptor complex involves a RLP named RBPG1 and its co-receptor the RLK SOBIR1 (Zhang et al., 2014) and given the role of SOBIR1 as important co-receptor of many RLPs (Gust and Felix, 2014), the investigation of its grapevine ortholog definitively deserves attention. Lastly, the grapevine genome contains at least one clear orthologue gene of the OG receptor WAK1, which is induced during infection of grapevine flowers with *B. cinerea* (Haile et al., 2017). For all these PRR candidates, the development of genome editing by CRISPR-Cas9 could be a very useful technology to test the function of these putative orthologs through a loss-of-function approach in grapevine. The recent evidences that this technology can be efficiently used in this crop opens promising perspectives (Wang et al., 2018).

**Figure 3:**
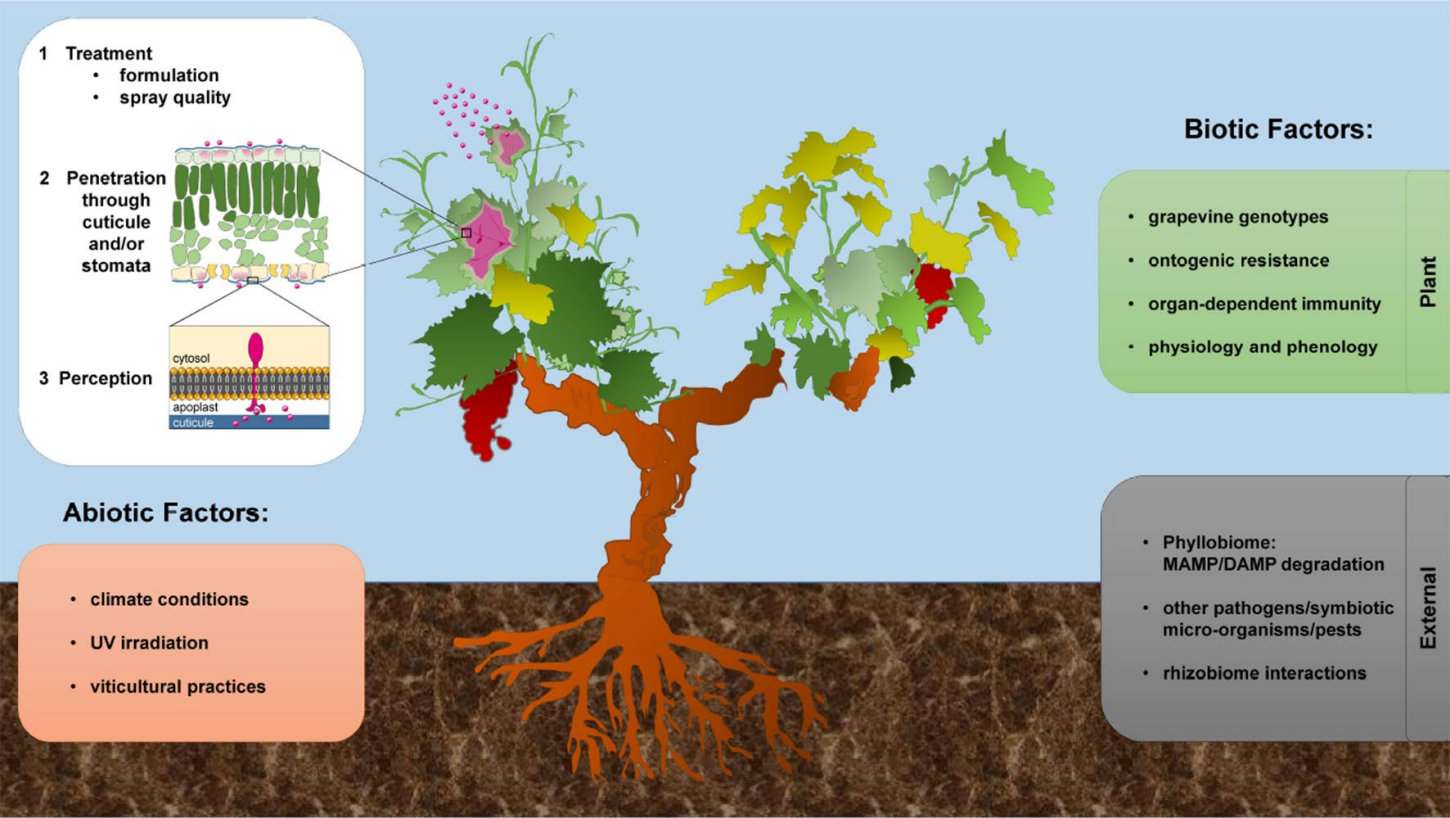
Effect of different factors influencing grapevine induced resistance in vineyards.

This fundamental knowledge will be important to better apply MAMPs/DAMPs to induce grapevine resistance, particularly in vineyards. Indeed, some biocontrol products are now homologated in the vineyards, such as the COS-OGA (van Aubel et al., 2014). However, are these elicitors actually perceived by the plant in natural conditions? This open question is of particular importance considering a perennial crop, which will require a deep investigation of the expression of the different immune receptors at different phenological stages and in different organs and taking plant aging into account. Since PRR-mediated immune responses vary during plant development (Zou et al., 2018), it would be interesting to know for instance if VvLYK1 and VvWAK1, two receptors involved in COS-OGA perception, are expressed in young leaves and flowers, two organs that are highly susceptible to filamentous microbes (**Figure 3**). Moreover, we must keep in mind the importance of plant genotypes (**Figure 3**) as the grapevine cultivars can differently respond to treatment with the same resistance inducers (Banani et al., 2014). In *Arabidopsis*, it has been shown that the existing natural mutations are sufficient to explain that some genotypes are totally unable to sense MAMPs as flg22 (Felix et al., 1999; Zipfel et al., 2004; Vetter et al., 2012). This supports the need for a better knowledge of the molecular mechanisms to ensure a broad and durable action of elicitors as resistance inducers in grapevine.

### Searching for Effective IR Markers

Concerning MAMPs/DAMPs, the screening of active biomolecules must continue to identify new resistance inducers and develop their use in crop protection strategies. However, the stimulation of the immune system is not systematically correlated with IR. Hence, from an applicative point of view, it is necessary to identify effective IR markers. It was for this purpose that a comparative experiment was performed with two oligosaccharidic elicitors differing in their capacity of inducing resistance against *P. viticola*: one effective (PS3) and one non-effective (a short laminarin of DP 13 that triggers production of H2O2 without inducing any protection). On one hand, proteomics showed that only few proteins, including the 12-oxophytodienoate reductase (OPR-like) related to the OPDA pathway and an arsenite-resistance protein (serrate-like protein), were found as possible markers of PS3-IR (Lemaître-Guillier et al., 2017). On the other hand, metabolomics analysis highlighted erythritol phosphate, a phosphorylated polyol, as another possible marker of PS3-IR. In parallel, the microarray analysis showed that the PS3-induced transcriptomic profile includes genes encoding a SA methyl transferase and terpene synthases (Gauthier, 2009; Gauthier et al., 2014). Further investigation of the emission of volatile organic compounds (VOCs) in grapevine plants treated with PS3 showed the production of mono- and sesquiterpenes, notably trans-β-ocimene and (E-E) α-farnesene (Chalal et al., 2015). Altogether, these experiments revealed a rather limited number of putative IR markers, and we cannot rule out the possibility that IR markers would differ with other resistance inducers or in other genotypes. It will therefore be probably more reasonable to consider “metabolic signatures” of effective resistance markers rather than a specific one. In this context, the non-destructive monitoring of VOCs may become a convenient way to study the plant response to resistance inducers in the field.

### Factors Influencing Efficient Grapevine IR in Vineyards

Polysaccharides and lipids are particularly interesting because many of them are approved for human feeding, and their costs of production are compatible with an application in viticulture. However, in field conditions, it is clear that only few elicitors, proved to be effective to induce resistance in controlled conditions, maintain their performances (Delaunois et al., 2014). Besides the above-mentioned lack of knowledge of elicitor recognition in natural conditions, the effectiveness of elicitor induced-resistance depends also on several biotic and abiotic factors (**Figure 3**). First, the active molecules must penetrate the plant tissues to elicit innate immunity. For oligosaccharidic elicitors (e.g., laminarin or PS3), the main barrier is represented by the cuticle, which is difficult to cross due to high hydrophobicity, and also generally thicker in field conditions. Using a fluorescently tagged PS3, it was shown how compound formulation is important for improving the bioavailability of certain elicitors (hydrophilic) and, consequently, their effectiveness to induce resistance (Paris et al., 2016). Such works also showed that the stomata, present in grapevine almost exclusively on the underside of the leaves, represent an important entry route for these treatments. Finally, it is nevertheless possible to improve the penetration of hydrophilic elicitors by (i) an *ad hoc* formulation with surfactants, and/or (ii) targeting a spray on the more permeable lower leaf surface (**Figure 3**).

The stability of the applied elicitors is another understudied aspect to consider for plant protection. Indeed, elicitors could be degraded after application, notably by plant microbiome (Trouvelot et al., 2014). For instance, laminarin is a substrate for β-1,3-glucanases. Thus, a basal activity of microbial or plant glucanases could degrade laminarin and, consequently, releases short inactive β-glucans. This biodegradation of elicitors might be very rapid in natural conditions and may partly explain the lower efficiency of these resistance inducers in vineyards (**Figure 3**). Interestingly, laminarin sulfation clearly protects the molecule from its enzymatic degradation, thus increasing its stability (Ménard et al., 2005) and probably explaining the higher resistance induced by PS3 compared to laminarin (Gauthier et al., 2014). In the same way, plant enzymes such as the BBE-like proteins play a crucial role in controlling the elicitor activity of oligosaccharides. According to their oxidase function dampening the excessive accumulation of active OGs and CDs (Benedetti et al., 2018; Locci et al., 2019), we could assume that such plant enzymatic activities may interfere with oligosaccharide stability as active elicitors. Moreover, in *Arabidopsis*, BBE-like proteins constitute a family of 28 members, and so far, only 5 of them have been demonstrated to be involved in the DAMP-homeostasis (OGs and CDs). The remaining uncharacterized members of this family might be responsible for the inactivation of “orphan” cell-wall-derived DAMPs, such as xyloglucans or the less characterized “Burdock” fructo-oligosaccharides (Sun et al., 2013). The identification of the BBE-like protein family in *Vitis vinifera* should also be investigated for a better knowledge of oligosaccharide defense induction in grapevine and their possible application. Other environmental factors must also be taken into account, such as molecule degradation by intense UV light, washing by rain falls or a rapid drying preventing the penetration of the elicitors through the cuticle (**Figure 3**). Therefore, the formulation of these new biocontrol products must be optimized to ensure molecule stability in changing conditions and allow their rapid penetration through cuticle and cell wall.

Finally, many other factors, such as viticultural practices or interactions with the plant-associated microbiomes, could impact the plant physiology which is determinant to mount an efficient IR in field conditions.

## Author Contributions

M-CH, MA, DB, JC, SC, XD, SD, AG, CL-G, JN, LT, ST, EV and BP contribute together to the writing of this review.

## Funding

M-CH, MA, DB, JC, SC, XD, SD, AG, CL-G, JN, LT, ST, EV and BP have been financially supported by ANR, Feder, the Regional Council of Bourgogne Franche-Comté and the Bureau Interprofessionnel des Vins de Bourgogne. SD, SC, ST, M-CH, CL-G, MA and BP have been supported by the MESRI (Ministère de l’Enseignement Supe´rieur, de la Recherche et de l’Innovation).

## Conflict of Interest Statement

The authors declare that the research was conducted in the absence of any commercial or financial relationships that could be construed as a potential conflict of interest.
